# Enhanced accuracy through machine learning-based simultaneous evaluation: a case study of RBS analysis of multinary materials

**DOI:** 10.1038/s41598-024-58265-7

**Published:** 2024-04-08

**Authors:** Goele Magchiels, Niels Claessens, Johan Meersschaut, André Vantomme

**Affiliations:** 1 Quantum Solid-State Physics, KU Leuven, Celestijnenlaan 200D, 3001 Leuven, Belgium; 2 IMEC, Kapeldreef 75, 3001 Leuven, Belgium

**Keywords:** Surfaces, interfaces and thin films, Characterization and analytical techniques

## Abstract

We address the high accuracy and precision demands for analyzing large in situ or *in operando* spectral data sets. A dual-input artificial neural network (ANN) algorithm enables the compositional and depth-sensitive analysis of multinary materials by simultaneously evaluating spectra collected under multiple experimental conditions. To validate the developed algorithm, a case study was conducted analyzing complex Rutherford backscattering spectrometry (RBS) spectra collected in two scattering geometries. The dual-input ANN analysis excelled in providing a systematic analysis and precise results, showcasing its robustness in handling complex data and minimizing user bias. A comprehensive comparison with human supervision analysis and conventional single-input ANN analysis revealed a reduced susceptibility of the dual-input ANN analysis to inaccurately known setup parameters, a common challenge in material characterization. The developed multi-input approach can be extended to a wide range of analytical techniques, in which the combined analysis of measurements performed under different experimental conditions is beneficial for disentangling details of the material properties.

## Introduction

Accurate characterization of complex planar and 3D structures is, amongst others, essential for successful device fabrication and implementation in micro- and nanotechnology^1^. The state-of-the-art device fabrication involves various annealing processes, including thermal annealing for creating two-dimensional multilayered structures, and thermally activated topography annealing or pulsed laser annealing for shaping three-dimensional structures^2^.

The (heat-induced) change in structural, electrical, and chemical properties throughout the fabrication process is very often probed using in situ and *in operando* techniques^3,4^. In these approaches, a measurement is continuously repeated to discern the evolution of the sample properties *during* the processing, providing a large data set that allows one to monitor subtle changes between consecutive steps.

Nonetheless, the analysis of the resulting large data set requires a rapid and systematic method for examining each measurement step. Recent studies have demonstrated the successful deployment of machine learning algorithms for augmentation and high-throughput analysis of data generated by a wide variety of experimental techniques, aiming to extract valuable structural and topographical information^5–11^. These include nuclear resonant scattering of synchrotron radiation, reflective high-energy electron diffraction, X-ray diffraction, and Rutherford backscattering spectrometry (RBS)^12–17^. So far, machine learning algorithms have focused on analyzing data sets obtained by a single experimental technique within a single geometry. However, a more advanced and comprehensive understanding can be achieved by employing machine learning to simultaneously analyze data collected from various measurement geometries or conditions, or by integrating data from different experimental techniques^18^.

As an example, RBS enables multi-geometry data collection through the scattering of incident ions with the atoms of the target material, followed by the measurement of the energy of the backscattered ions at various scattering angles. This technique enables a high throughput of data, absolute yield quantification (without calibration standards), and absolute depth resolution, therefore making RBS propitious for real-time studies and the detection of (minor) structural changes such as layer thickness of multilayered materials, layer roughness and porosity, nucleation of a new stoichiometric phase, elemental diffusion, etc^19–24^. The compositional depth profile of the target can be directly derived from the measured spectrum via three quantities: (1) The *atomic mass* of the target atoms is obtained via the so-called kinematic factor, i.e., the ratio of the ion energy after and before scattering, and the kinematic factor increases with increasing target atomic mass; (2) The *elemental concentration* at a particular depth is obtained by the yield of detected backscattered ions at the corresponding energy; (3) The *depth information* is obtained via the width of the elemental signal and the shift in energy of elemental signals originating from deeper within the sample, caused by (small-angle) collisions of the incoming ions with target electrons (electronic stopping).

**Figure 1:**
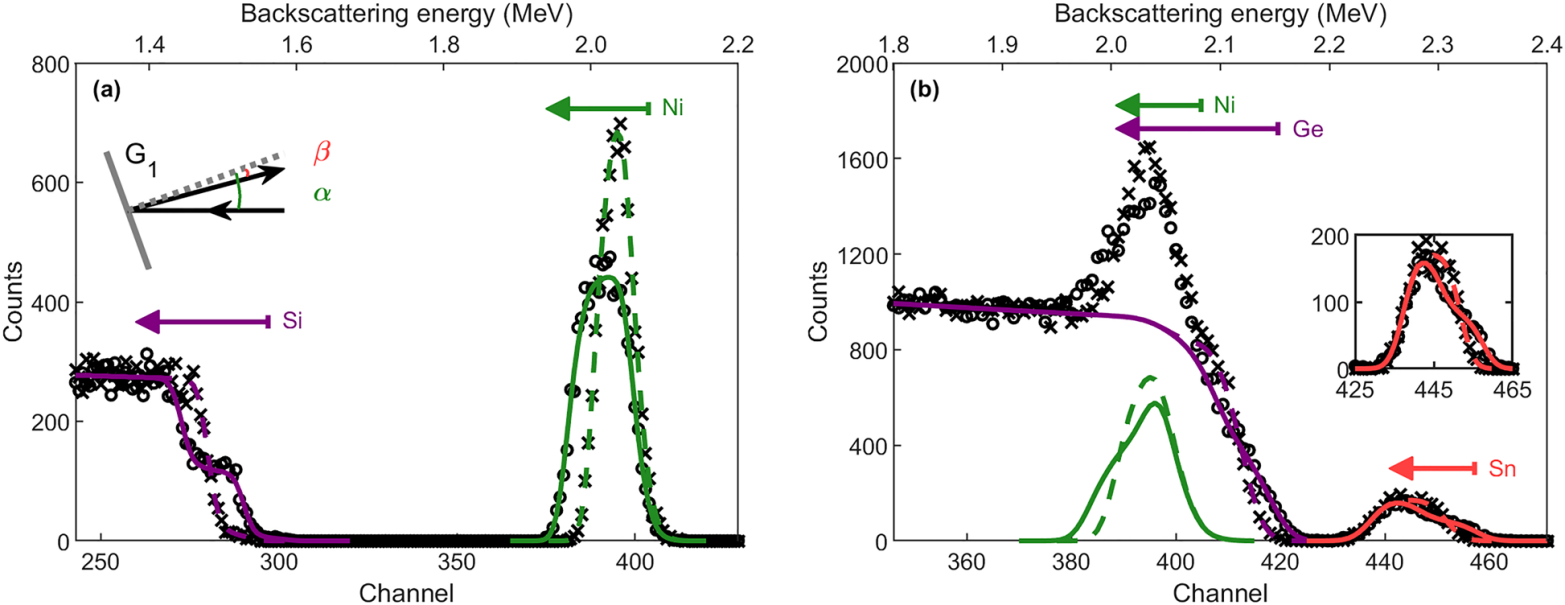
Simulated (including Poisson statistics) RBS spectra of (**a**) a 48 nm Ni/Si bilayer (black crosses) and a 137 nm NiSi/Si bilayer (black open circles) and (**b**) a 48 nm Ni/117 nm Ge_1−_Sn/Ge multilayer (black crosses) and a 22 nm Ni/49 nm Ni_5_(Ge_1−_Sn)_3_/40 nm NiGe_1−_Sn/73 nm Ge_1−_Sn/Ge multilayer (black open circles) with x=0.08, using a 2.7 MeV He^2+^ beam and detection in the G_1_ geometry, as shown in the inset in (**a**). The Ni (green), Si and Ge (purple, in (**a**) and (**b**), resp.) and Sn (red) contributions to the RBS spectra are highlighted. The arrows indicate the respective elemental depth profiles, starting from the sample surface. The inset in (**b**) illustrates the difference in the Sn signal for the two spectra.

RBS is ideally suited for the study of multilayered materials with sufficiently different atomic masses (hence sufficiently different kinematic factors), allowing energy separation of the elemental signals in the spectrum. A textbook example is the RBS analysis of a Ni film on a Si substrate, compared to a NiSi film on Si that is obtained after annealing the sample, as shown for the simulated spectra in Fig. 1a. On the one hand, in the spectrum emerging from the Ni/Si configuration, the low-energy (below $\sim$1.6 MeV), low-yield signal results from scattering in the Si substrate (1: atomic mass, as described above), whereas the high-energy (around $\sim$2.1 MeV), high-yield peak results from the Ni film (1: atomic mass). The width of the Ni peak, determined by electronic stopping, corresponds to the thickness of the film (3: depth information). On the other hand, in the spectrum emerging from the NiSi/Si configuration, a step in the Si signal can be observed as a result of the adjacent sub-signals arising from scattering with the Si substrate atoms (E < 1.45 MeV) and with the Si atoms in the NiSi film (E > 1.45 MeV). The energy width of the NiSi sub-signal, as well as the leading edge energy of the substrate sub-signal, are determined by the energy loss of the incoming ions in the NiSi film (3: depth information). The ratio of the sub-signal yields directly reflects the ratio of the Si concentrations in the respective layers (1 in substrate, 0.5 in NiSi layer) (2: elemental concentration). Concomitant with the changes in the Si signal, the Ni signal exhibits broadening and decreased yield as a result of the NiSi layer thickness and composition (1:1 ratio of Ni and Si atoms). Notably, the integrated Ni signal in the RBS spectra of both sample configurations remains constant, implying conservation of the number of Ni atoms within the system.

The conventional approach to deduce the compositional depth profile from the RBS measurement is by spectrum fitting varying the sample parameters and using a forward simulator^25,26^. Whereas the forward simulation of a defined target results in a uniquely defined RBS spectrum, the inverse problem of finding the compositional depth profile from experimental RBS data can be more ambiguous.

Highly reliable solutions (compositional depth profiles) for spectra within a real-time data set can be obtained by employing Butler’s three criteria, even though such solutions may not possess mathematical uniqueness^27^. The first criterion is conservation of mass, which implies conforming to conservation of the total areal density of elements present in the target. The second criterion is adherence to thermodynamic principles, which implies conforming to thermodynamically stable phase stoichiometries formed by annealing. The third and foremost criterion for this study is the combined evaluation of spectra, which are collected in multiple scattering geometries. This can be done either in an *iterative* way, i.e., sequentially, one spectrum’s analysis begins with an initial assumption derived from another spectrum’s analysis result, or in a *simultaneous* way, i.e., a direct approach in which a parameter is fitted to multiple spectra at the same time^28^. However, both the iterative and simultaneous approach are time-demanding, making them suboptimal for analyzing large quantities of data. Moreover, in both approaches, the operator is free to impose different weights on the contribution of each spectrum to the final compositional depth profile, resulting in a user-biased analysis.

The user bias can be minimized by applying a machine learning approach. The latter mainly involves artificial neural networks (ANN), which relate a single RBS spectrum to the corresponding compositional depth profile^12,29,30^. Analogously to the transmission and processing of electric signals in a biological network of neurons, the architecture of a multilayer perceptron ANN consists of one layer of input nodes and one layer of output nodes, separated by one or more hidden layers. The information is transmitted from the input to the output layer by a forward, fully interconnected network of nodes. A nonlinear activation function is applied to the weighted sum of the nodes in one layer, resulting in the value of the node in the next layer. The weight value of each interconnection is determined by the learning of the ANN using a training set consisting of established input-output patterns, and the iterative adaption of weights to minimize the mean-square error on the outputs of the test set—an approach known as supervised learning. Thereafter, the trained ANN allows extremely fast analysis of large sets of data within the parameter space defined by the training set. This parameter space encompasses a multidimensional range of values for the target and RBS setup parameters, used for the generation of the ANN training set.

Unlike forward fitting, this machine learning-based approach lacks any prior knowledge of the physics underlying the experiment (here: Rutherford scattering). Until now, the focus of machine learning analysis approaches has primarily been on large data sets of single geometry RBS data. While a previous study suggested the potential of combining the analysis of multiple RBS and elastic recoil detection analysis measurements within a single ANN, the work did not investigate the actual capabilities of this approach, as it tackled a straightforward problem that did not necessitate simultaneous analysis^31^.

This study establishes a multi-input ANN algorithm for the analysis of complex RBS data sets. This algorithm simultaneously relates spectra measured in multiple scattering geometries to a unique compositional depth profile. We show the advantages, limitations, and pitfalls of the dual-input ANN analysis by applying this newly developed approach to the real-time RBS data set of the thermal reaction of Ni with Ge_1-x_Sn_x_^32^. The complexity of this data set surpasses previously studied cases, presenting an exceptional level of challenge that reaches the limits of conventional analysis approaches. Therefore, a simultaneous and non-user-biased analysis is required to minimize ambiguity.

### Introduction to the Ni-Ge_1-x_Sn_x_ data set

The Ni-Ge_1-x_Sn_x_ thermal reaction data set was collected in the scope of a study of Ni-germanide formation in the presence of strained Ge *channels*, which were introduced in microelectronics to enhance the hole mobility. One way to induce strain is by alloying a fraction of Sn to Ge, resulting in a lattice mismatch between Ge_1-x_Sn_x_ and the Ge substrate^33–35^. For pure Ge, it was demonstrated that the thermally-induced NiGe phase exhibits exceptional contact properties on Ge, surpassing those of other transition metal germanides^36^. Based on this finding, the thermal reaction of a thin Ni film with Ge_1-x_Sn_x_ was studied by Demeulemeester et al.^32^. To understand the influence of the alloying of Sn on the phase sequence and on the reaction kinetics, real-time RBS was applied, i.e., continuously capturing RBS spectra while the thermal reaction of Ni with Ge_1-x_Sn_x_ takes place. At annealing temperatures up to 300 °C, referred to as the low-temperature domain, the same phase sequence was observed as for Ni/Ge (Ni/Ge $\rightarrow$ Ni_5_Ge_3_ $\rightarrow$ NiGe), including a constant Sn fraction in the formed germanides. At annealing temperatures exceeding 300 °C, referred to as the high-temperature domain, Sn redistribution in the NiGe_1-x_Sn_x_ phase occurred.

**Figure 2:**
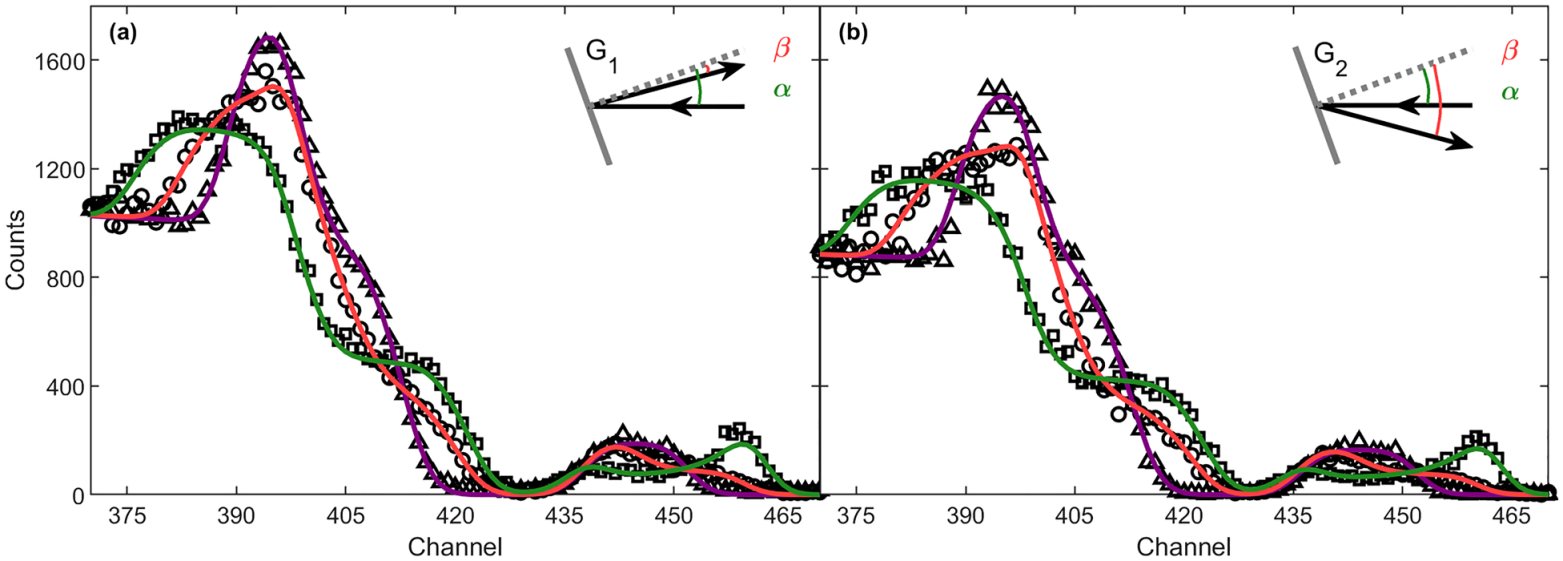
Experimental (black data points) and simulated RBS spectra (solid lines) based on the dual-input ANN analysis in (**a**) the G_1_ geometry and (**b**) the G_2_ geometry (for T = 32 °C (triangles, purple), 246 °C (circles, red), 402 °C (squares, green).

The RBS measurements were performed on the Ni/Ge_1-x_Sn_x_/Ge multilayer using an incident beam of 2.7 MeV He^2+^ ions that scattered from the sample, which was mounted at a tilt angle $\alpha$ of 20°. The scattered particles were simultaneously detected at exit angles (i.e., the angle between the surface normal of the sample and the detected outgoing beam) of $\beta _1~=~5$° and $\beta _2~=~35$°. These geometries will be referred to as G_1_ and G_2_, respectively (see inset in Fig. 2). RBS spectra were acquired while the annealing temperature applied to the multilayer was ramped between room temperature and 430 °C, resulting in a data set of 80 spectra per detection geometry at a collection rate of 4 °C per measurement (except for the fast ramp up to 150 °C).

Figure 1b shows the simulated RBS spectrum using SIMNRA for an as-deposited Ni/Ge_1-x_Sn_x_/Ge sample and a multilayer of thermodynamically stable Ni germanide phases induced by thermal annealing for the G_1_ scattering geometry. Several complexities emerge from these spectra. First, as a result of the higher atomic mass of Ge compared to Ni and the electronic stopping of the incoming He^2+^ ions in the Ni layer, the Ge high-energy edge and the Ni signal are superimposed in the RBS spectrum (purple and green signals). Second, upon thermal reaction, the real-time study comprises spectra with the simultaneous presence of the unreacted Ni, Ni_5_(Ge_1-x_Sn_x_)_3_, and NiGe_1-x_Sn_x_ phases. This coexistence of multiple very thin layers with varying thicknesses and composition, in combination with the superimposed Ni and Ge signal, results in non-unique solutions to the compositional depth profile. Third, the small Sn yield results from the small Sn concentration ($x = 8\%$), which complicates the probing of the Sn redistribution occurring at elevated temperatures. Considering these complexities and ambiguities, this data set is well suited to explore and push the boundaries of simultaneous multi-detector ANN analysis for highly convoluted RBS spectra. When assessing the network capabilities, the human supervision analysis performed by Demeulemeestrer et al.^32^ serves as a benchmark for our results. This human supervision analysis involves the iterative fitting of the measurements in the G_1_ and G_2_ scattering geometry by sequentially using the analysis of the RBS spectrum in one geometry as input for the analysis in the other geometry.

## Results

In the scope of this study, three ANN analysis algorithms were developed: a single-input ANN analyzing the G_1_ scattering geometry (SI-G_1_) and the G_2_ geometry (SI-G_2_), and a dual-input (DI) ANN. A comparative assessment of their analysis accuracy, precision, and reliability is conducted, benchmarking against both each other and the human supervision analysis.

### Multi-detector artificial neural network

Ideally, one dual-input ANN is designed and trained for simultaneous evaluation of the G_1_ and G_2_ scattering geometry measurements of the entire RBS data set; however, the generality of the parameter space fully covering the phase formation as well as the Sn redistribution results in a decreased accuracy of the ANN^37,38^. Because of this generality-precision trade-off, two distinct dual-input ANNs were designed and trained to cover the two physical processes occurring during the thermal reaction. First, the low-temperature domain ANN (DI, SI-G_1_, SI-G_2_) models the growth and consumption of the unreacted Ni layer, the Ni_5_(Ge_1-x_Sn_x_)_3_ phase and the NiGe_1-y_Sn_y_ phase, preserving a uniform Sn distribution in each phase, and Sn enrichment at the surface and interfaces. Second, the high-temperature domain DI ANN describes the redistribution of the alloying Sn in the final NiGe_1-y_Sn_y_ phase and Sn enrichment at the surface and interfaces.

The architecture of both DI ANNs is a multilayer perceptron, whose inputs comprise the counts per channel within the region of interest of two simultaneously measured RBS spectra. For both scattering geometries, the region of interest consists of the channels encompassing the Ni, Ge, and Sn signals after normalization to the Ge substrate counts. The ANN outputs are the areal densities of the elements in the respective layers (*Nt*, i.e., the product of the atomic density *N* and the film thickness *t*, hence directly related to the number of atoms).

Supervised learning of the ANNs (low- and high-temperature domain DI, SI-G_1_, SI-G_2_) was applied. The training set consisted of patterns of randomly selected compositional depth profiles and the corresponding RBS spectra in scattering geometries G_1_ and G_2_, simulated using SIMNRA^39^. The compositional depth profiles were generated by randomly selecting the areal densities of the elements in the respective layers from a normal distribution. The only free setup parameter is the energy calibration offset, allowing the validation of the ANN analysis if a gradual spectrum shift occurs during the long real-time run. Such shifts may originate from various factors, including minor drift in the data acquisition electronics and ion beam-induced carbon deposition^40^.

Following the supervised learning, the relative contribution of the inputs to the individual output nodes can be understood using Garson’s algorithm or activation maps^41,42^. Applying Garson’s algorithm to the low- and high-temperature domain ANN showed that the RBS spectra from both scattering geometries G_1_ and G_2_ contributed substantially to the ANN output. To obtain the accuracy and precision of the low- and high-temperature domain ANN analysis, ten ANNs are trained independently using the same training set and employed for data analysis. For each dual-spectrum input, this analysis with the independently trained ANNs results in the mean value and standard deviation of each ANN output^30^.

### Feasibility and results of dual-input ANN analysis

The dual-input ANN analysis of the experimental spectra provides the mean areal densities of the elements in the respective layers (i.e., compositional depth profile) at each temperature step. Subsequent *simulation* of the RBS spectra based on the obtained compositional depth profile is performed using SIMNRA. These simulated spectra are superimposed with the experimental spectra to demonstrate the accuracy of the dual-input ANN analysis. It should be emphasized that the simulations are exclusively normalized to the Ge substrate yield and fitted to the experimental spectra by varying the energy calibration offset. The latter is valid as the offset is a free parameter in the training set. In particular, no further adjustment of the sample parameters is made, in contrast to what is often found in literature where a subsequent *fitting* step is applied post-ANN analysis. As an example, the experimental data measured at an annealing temperature of T = 32 °C, 246 °C, and 402 °C, superimposed with the corresponding simulations based on the DI ANN analysis are shown in Fig. 2 for both scattering geometries.

**Figure 3:**
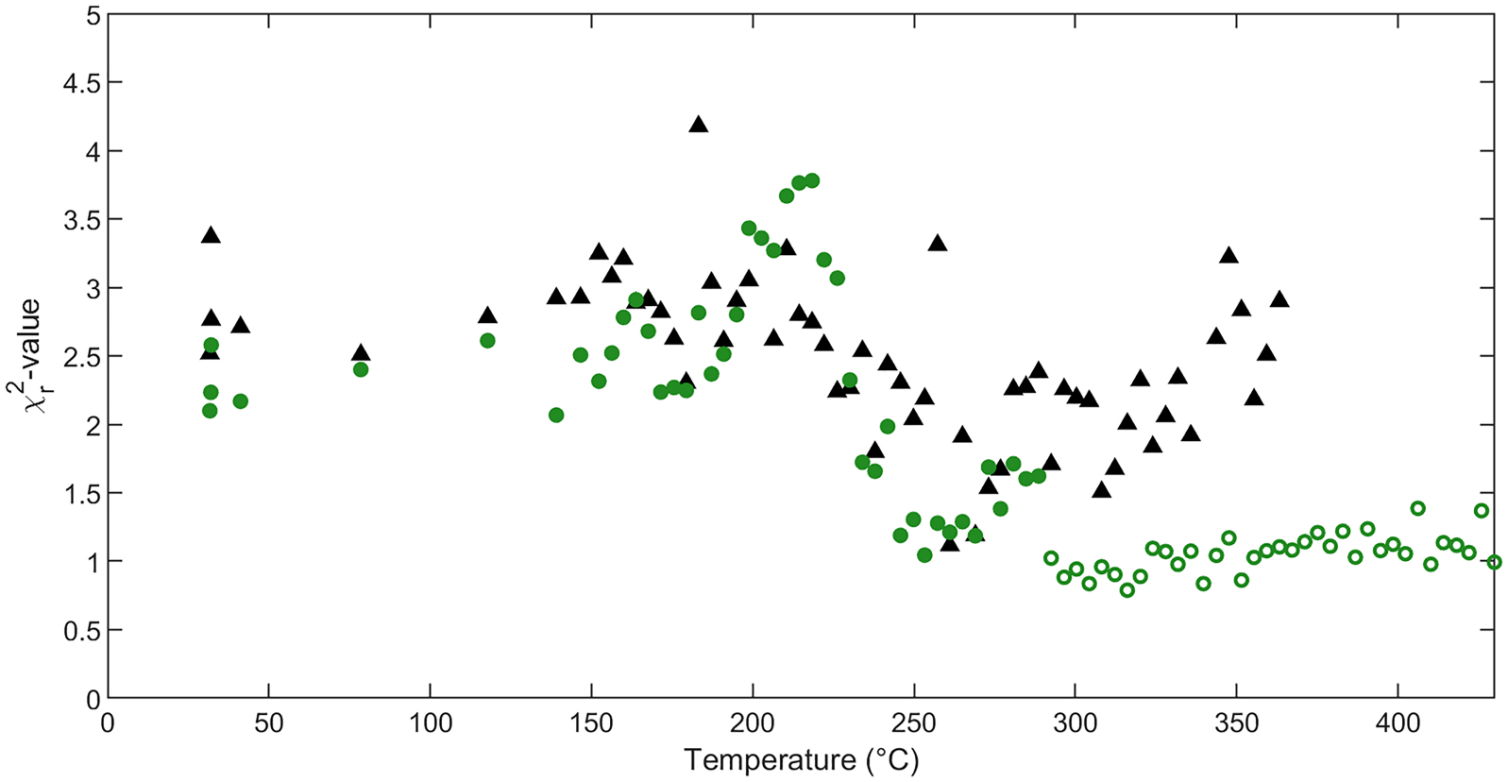
Evaluation of reduced quadratic deviation of the spectra in the G_1_ geometry after dual-input ANN analysis (low-temperature domain: green filled circles, high-temperature domain: green open circles) and after human supervision analysis (black triangles).

The high accuracy is evidenced by the excellent agreement between the experimental and the simulated spectra throughout the entire low- and high-temperature domain. Next, the reduced-square deviation between the experimental and simulated data ($\chi ^2_r$-value, i.e., $\chi ^2$ divided by number of channels) is calculated for each measurement and plotted as a function of annealing temperature (Fig. 3). The $\chi ^2_r$-values resulting from the low-temperature domain DI ANN analysis are comparable to the human supervision analysis, confirming the accurate dual-input ANN analysis. The $\chi ^2_r$-values of the high-temperature domain DI ANN analysis are considerably smaller than those of the low-temperature domain DI ANN analysis, arising from the reduced level of complexity in the high-temperature domain DI ANN training set.

**Figure 4:**
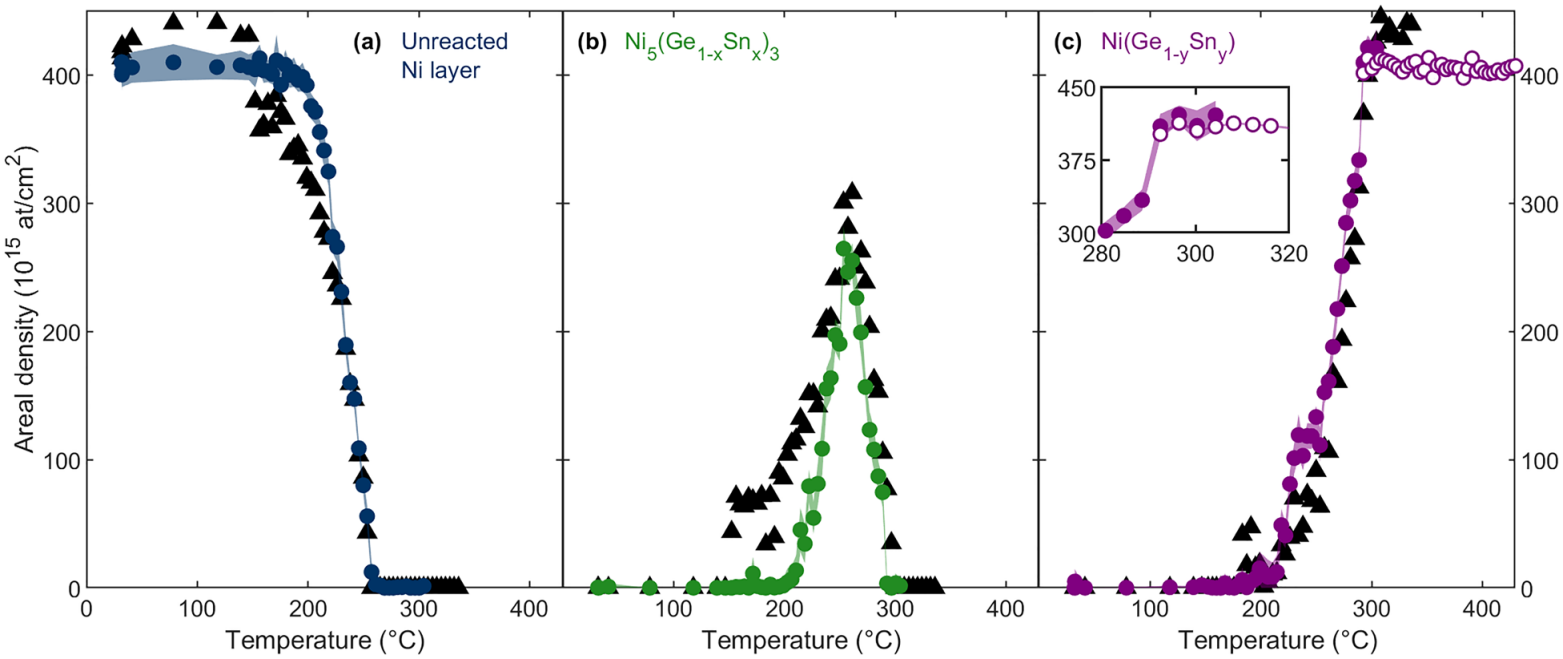
Ni areal density as a function of temperature of the unreacted Ni layer (**a**, blue), Ni in the Ni_5_(Ge_1-x_Sn_x_)_3_ phase (**b**, green), and Ni in the NiGe_1-y_Sn_y_ phase (**c**, purple) obtained by dual-input ANN analysis (data points correspond to the mean areal density, error band to the uncertainty covering 1 $\sigma$, acquired through the analysis using ten independently trained ANNs), and the corresponding human supervision analysis (black triangles). The inset in (**c**) compares the low-temperature domain (filled symbols) and high-temperature domain (open symbols) dual-input ANN analysis for NiGe_1-y_Sn_y_ in the overlapping temperature range.

The comparison of the phase formation and consumption between the analysis by the dual-input ANN and by human supervision is given in Fig. 4. The DI ANN analysis indicates a higher onset temperature for the growth of the Ni_5_(Ge_1-x_Sn_x_)_3_ phase, concomitant with a higher onset temperature for the consumption of the Ni surface layer (Fig. 4a,b). This difference in the onset temperature of thermal reaction discloses the necessity for *simultaneous* analysis (in contrast to *iterative* human supervision analysis applied before) and the susceptibility of the human supervision analysis to user bias, as will be discussed in Section ‘Iterative versus simultaneous analysis’. In addition, the human supervision and DI ANN analysis approach agree in the prediction of the total phase consumption temperature for both the unreacted Ni layer and the Ni_5_(Ge_1-x_Sn_x_)_3_ layer. In the temperature range between 292 and 304 °C (i.e., the transition from the low- to the high-temperature domain), the two DI ANNs provide consistent results. This consistency affirms the high precision and reliability of the low- and the high-temperature domain DI ANN at the respective high- and low-temperature limits of their parameter space (Fig. 4c). Moreover, this indicates that the same result is obtained independently of the differently modeled systems (phase growth vs. Sn redistribution).

**Figure 5:**
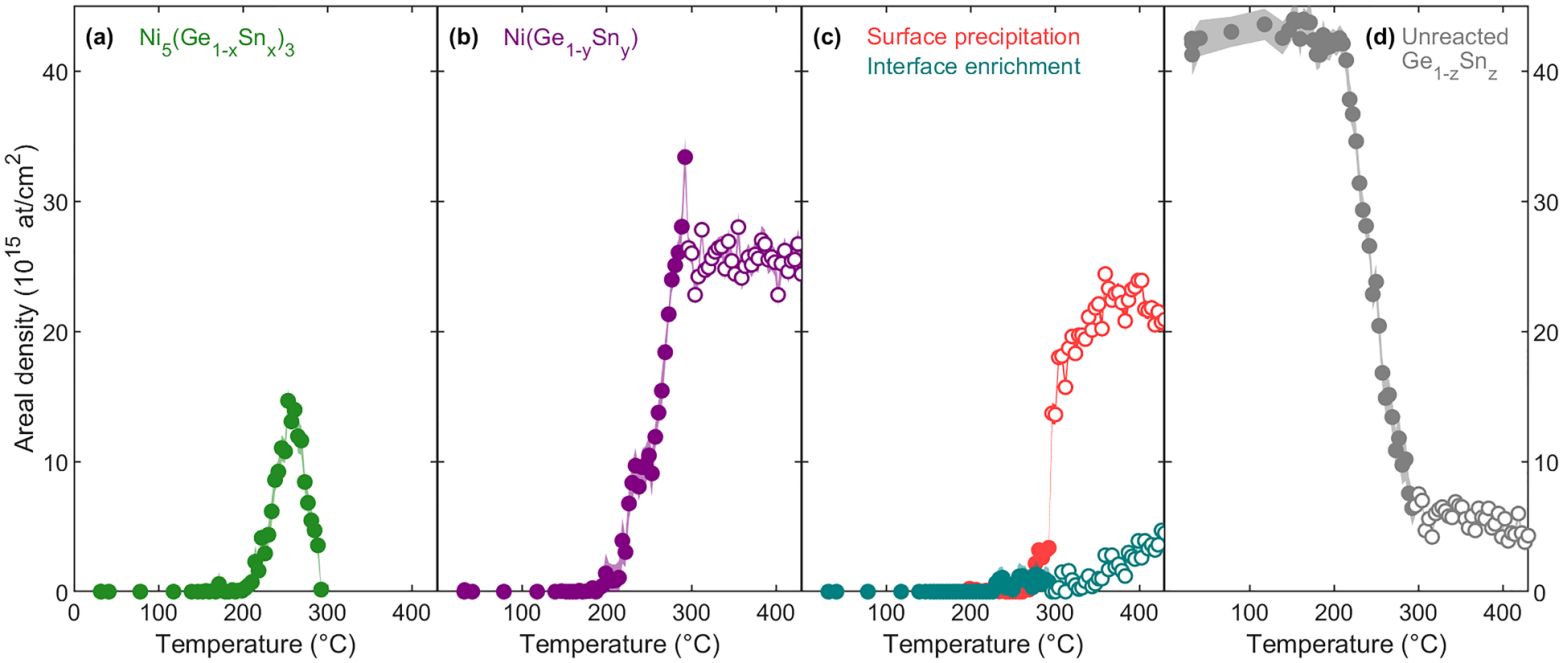
Sn Areal density as a function of temperature of Sn in the Ni_5_(Ge_1-x_Sn_x_)_3_ phase (**a**, green), the NiGe_1-y_Sn_y_ phase (**b**, purple), the surface precipitation (**c**, red), the germanide-Ge_1-z_Sn_z_ interface layer (**c**, teal), and the unreacted Ge_1-z_Sn_z_ layer (**d**, gray) obtained by dual-input ANN analysis, together with the analysis uncertainty covering 1 $\sigma$. The low-temperature domain (filled symbols) and high-temperature domain (open symbols) results are shown.

Furthermore, Butler’s three criteria for the ambiguity reduction of RBS analysis are fulfilled by this simultaneous evaluation approach using machine learning: (1) Conservation of the total Ni areal density is obtained for both the low- and high-temperature domain DI ANN, even though the total Ni areal density was varied in the training set within a normal distribution ($\mu$ = 220$\times 10^{15}$ atoms/cm^2^, $\sigma$ = 110 $\times 10^{15}$ atoms/cm^2^); (2) The ratio of the Ni (Fig. 4), Ge, and Sn (Fig. 5) areal densities in each phase agrees with the expected thermodynamically stable stoichiometries, including a homogeneous Sn distribution in the low-temperature domain; (3) The quantification is obtained by simultaneous evaluation of RBS data measured in two scattering geometries.

The reduction in ambiguity that is obtained through the dual-input approach is particularly crucial when analyzing the Sn depth profile, given the limited yield of the Sn signal. The dual-input ANN analysis approach allows the deconvolution of the five contributions to the total Sn signal (Fig. 5). Conservation of the total Sn areal density was obtained for T < 300 °C. However, at higher temperatures, an apparent increase in the total Sn areal density was observed. Likewise, as the temperature increased, integrating the Sn-related raw counts in the experimental spectra indicated a similar increase in total Sn areal density, which would contradict the principle of total areal density conservation. Therefore, this observation of increasing total Sn areal density is not related to a breakdown of the DI ANN algorithm but rather to an unexpected artifact, which was presumably caused by the out-diffusion of mobile n-type dopants (Sb) from the Ge substrate, or alike^43^.

### Uncertainty of the dual-input ANN analysis

In addition to the statistical and systematic uncertainties, the uncertainty induced by the analysis itself must be included in the total uncertainty budget as well^44,45^. A common approach for the uncertainty evaluation of a trained ANN is through the analysis of a simulated test set covering the parameter space of the experiment. The ANN outputs are compared to the target composition, resulting in a prediction error^37^. However, unlike a simulated test set, experimental data are also susceptible to inaccurately known experimental parameters. Therefore, a simulated test set cannot be considered a valid representation to obtain the total uncertainty of the experimental data analysis. An alternative parameter to define the uncertainty as the $\chi ^2_r$-value obtained by comparison of the experimental data to the RBS simulation that is based on the ANN output, as shown in Fig. 3. However, this approach is strongly susceptible to ambiguity (i.e., multiple compositional depth profiles may result in an identical ‘fit’) and may lead to non-physical results.

Therefore, as proposed for SI ANN analysis of RBS spectra, the uncertainty was evaluated using a set of ten independently trained DI ANNs (identical architecture and training set)^30^. After the DI ANN analyses (ten networks) of the experimental data set, each output’s mean value and standard deviation ($\sigma$) were calculated. The examination of the standard deviation of the Ni areal density outputs as a function of temperature (indicated by the error band in Fig. 4) demonstrated the following characteristics: (1) Before thermal reaction occurs (low-temperature domain, 30 °C to 200 °C), the standard deviation of the unreacted Ni layer is 11 $\times \; 10^{15}$ atoms/cm^2^; (2) During thermal reaction (low-temperature domain, 200 °C to 300 °C), the standard deviation of Ni in the different phases remains constant at approximately 10 $\times \; 10^{15}$ atoms/cm^2^; (3) After completing the final NiGe_1-y_Sn_y_ phase (high-temperature domain, 300 °C to 430 °C), the standard deviation of Ni within the NiGe_1-y_Sn_y_ phase drops to 2 $\times \; 10^{15}$ atoms/cm^2^. Thus, the high-temperature domain ANN analysis exhibited a pronounced reduction in uncertainty compared to the low-temperature domain analysis, which can be attributed to the reduced complexity of the high-temperature domain training set. Furthermore, it confirms the advantage of dividing the entire experimental parameter space into subspaces (low-temperature domain, high-temperature domain) due to the generality-precision trade-off. Additional uncertainty reduction, at the cost of increased user bias, can be achieved by restricting the training set to a smaller parameter space, for example, by introducing conservation of the total areal density of specific elements in the training set.

### Iterative versus simultaneous analysis

Comparing the analysis conducted under iterative human supervision with the simultaneous dual-input ANN analysis reveals distinct trends in the evolution of the unreacted Ni areal density as a function of annealing temperature (Fig. 4a). This difference raises the question of whether the contrast between the iterative and simultaneous approaches, along with potential user bias associated with human supervision analysis, can explain the different temperatures at which the thermal reaction starts (see above).

**Figure 6:**
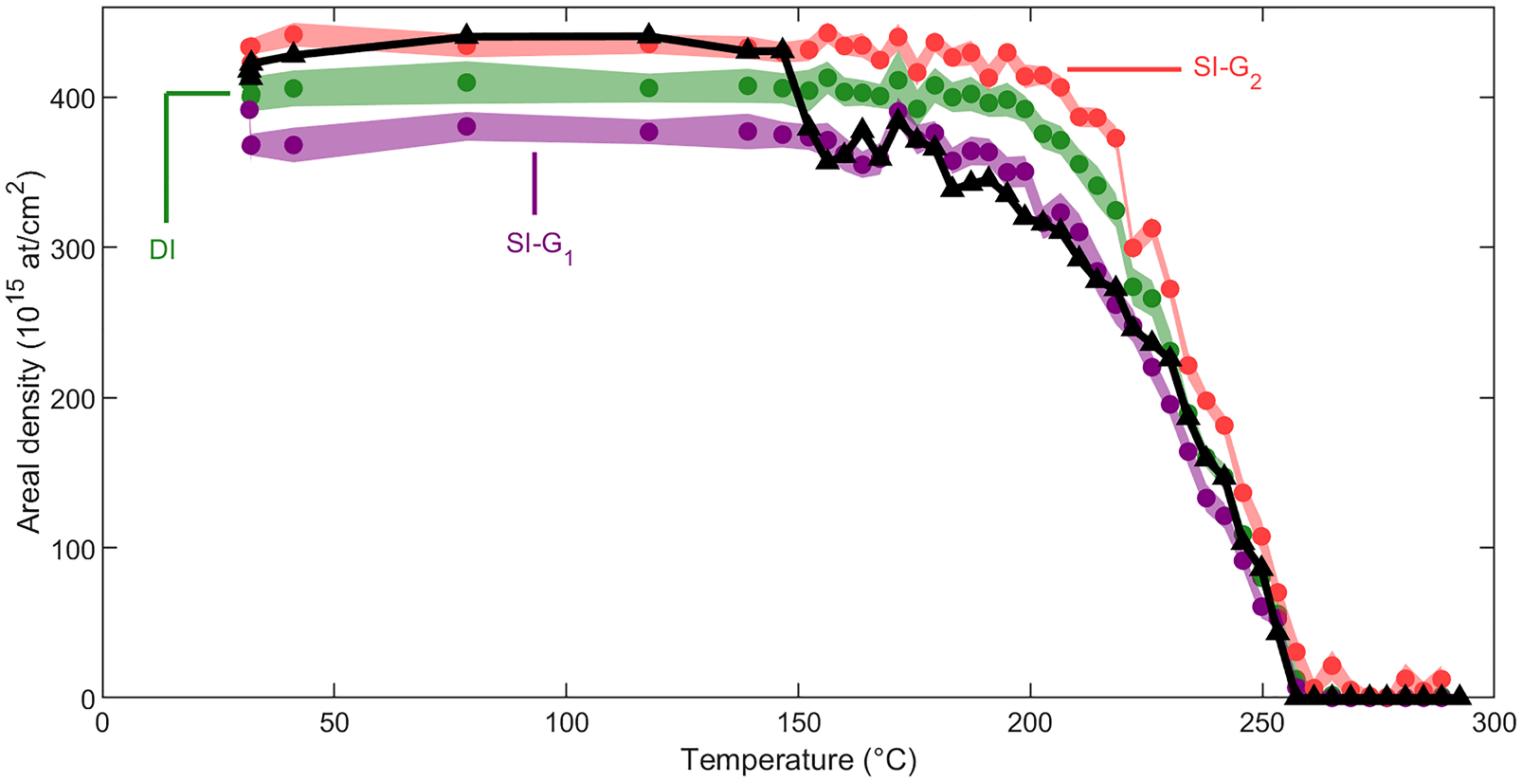
Ni areal density in the unreacted surface layer as a function of temperature, analyzed by the G_1_ single-input ANN (SI-G_1_: purple), the G_2_ single-input ANN (SI-G_2_: red), the dual-input ANN (DI: green), including the ANN analysis uncertainty covering 1 $\sigma$, and the human supervision analysis (black triangles).

In the pursuit of answers to this question, a set of single-input ANNs is trained (ten individual cycles of training, identical hidden layer architecture, and identical output nodes to dual-input ANN) for both the G_1_ (SI-G_1_) and G_2_ (SI-G_2_) geometry individually. The region of interest used as input of these ANNs is identical to that of the dual-input ANN. The resulting areal densities of the unreacted Ni layer are shown in Fig. 6. As the RBS measurements in the G_1_ and G_2_ scattering geometries were simultaneously performed, they originate from an identical target composition. Therefore, an accurate, unambiguous analysis should lead to identical areal densities independent of the scattering geometry. In contrast, a difference is noticed in the areal densities of the unreacted Ni layer following analysis with the SI-G_1_, SI-G_2_, and DI ANN approaches. Moreover, the standard deviations of the SI-G_1_ and SI-G_2_ ANN analysis are smaller than this areal density difference and, hence, can not explain the difference in the observed unreacted Ni areal density.

This raises questions regarding the accuracy of the setup parameters. Although it was attempted to determine the setup parameters with the highest accuracy possible, the potential for minor inaccuracies (minor deviations from the expected value) persists, which can result in systematic errors. For instance, any deviation in the detector position, and consequently, the scattering angle, changes the RBS spectrum. When the detector positions deviate in different directions (either positively or negatively) for either geometry, they have distinct effects on the spectra. Such inaccurately known detector positions could explain the difference in G_1_ and G_2_ analysis by SI ANNs. Finding the correct setup parameters through spectrum fitting is intricate due to the high correlation between the sample and setup parameters.

In contrast, when employing dual-input ANN analysis, minor deviations in setup parameters like beam energy, scattering angle, sample tilt, and energy-channel conversion (offset and gain) can be addressed by introducing an additional free setup parameter into the training set. Although each setup parameter affects the spectrum differently, in practice, at least for minor deviations, their effects can, to a first approximation, be modeled as spectrum shifts. Under this approximation, minor variations in the setup parameters are accounted for by including the free energy calibration offset within the training set (see “Multi-detector artificial neural network” section). Moreover, although it is possible to leave all setup parameters free, opting to vary only the offset substantially reduces the complexity of the training set and avoids highly ambiguous spectra, resulting in a more accurate analysis. Thus, the simultaneous dual-input ANN analysis provides a more robust approach by introducing a free spectrum shift into the ANN’s parameter space, effectively reducing sensitivity to inaccurately known setup parameters.

This robustness of the DI ANN approach outperforms conventional SI ANN analysis and human supervision analysis. On the one hand, the incapability of conventional SI ANNs in handling the analysis of spectra subjected to inaccurately known setup parameters is evidenced by the diverging results for SI-G1 and SI-G2, as shown in Fig. 6. This indicates that a reduction of the degree of freedom of the analysis is required to distinguish spectrum features related to sample composition from those arising due to slight deviations in setup parameters. One way to decrease the degree of freedom is by the combined analysis of RBS spectra collected in the two scattering geometries, ensuring a unique compositional depth profile that aligns with both spectra. The integration of this combined evaluation in the DI ANN enhances the reliability of the results compared to SI ANN analysis. On the other hand, human supervision analysis encounters difficulties in fitting complex spectra when attempting to adjust both the target parameters and the inaccurately known setup parameter. This challenge arises from the high correlation between these parameters. It can be addressed by constraining the possible solutions to the analysis (i.e., combinations of target and setup parameters), which allows the reduction of ambiguity in their correlation. This constrained parameter space is integrated into the DI ANN analysis through the training set. These considerations underscore the superior performance of the DI ANN analysis compared to both SI ANN analysis and human supervision ANN analysis.

Next, when comparing the areal density curves of the unreacted Ni layer (Fig. 6), it can be noted that the human supervision curve aligns with the SI-G_2_ curve between room temperature and 147 °C after which it jumps to the SI-G_1_ curve, overlapping until T = 226 °C. Above T = 230 °C, the human supervision curve transitions to the DI curve. These transitions in the *iterative* human supervision approach result from two instances of user bias in the analysis. First, changes in the relative weights imposed by the operator to the G_1_ and G_2_ contributions in the G_1_-G_2_ combined analysis across the temperature range of the real-time experiment lead to discontinuous jumps. Second, the operator may unintentionally be biased in imposing a trend to the areal density evolution. This occurs in two steps. Initially, it is the operator who decides when a new phase appears while the effect of the newly present phase on the spectrum is still minimal. Subsequently, the operator anticipates a systematic behavior in the presence and layer thickness evolution of a phase until the next phase emerges. Consequently, the shift from the SI-G_2_ to the SI-G_1_ curve during iterative human supervision analysis leads to an unintended underestimation of the onset temperature for phase formation (Fig. 4b). Moreover, the transitions of the human supervision analysis between the SI ANN curves suggest that the human supervisor could not identify a single, consistent compositional depth profile that aligns with both the G_1_ and G_2_ scattering geometries, conforming to the divergent SI ANN analysis. It demonstrates the sensitivity of iterative human supervision analysis to minor deviations from the expected setup parameters, leading to an inconsistent analysis. In other words, the human supervision analysis suffers from user bias despite the human supervisor’s effort to perform a self-consistent analysis. In contrast, the *simultaneous* DI ANN approach (not *iterative*) enables a systematic analysis throughout the entire temperature domain of the experiment.

## Discussion

A dual-input ANN algorithm has been developed to simultaneously evaluate RBS spectra measured in two scattering geometries. This analysis approach was applied to the large real-time RBS data set acquired during the thermal reaction of a Ni film with Ge_1-x_Sn_x_, which posed extensive challenges due to superimposed signals, adjacent thin layers containing varying element concentrations, and low concentrations. The accuracy of the dual-input approach was thoroughly examined by comparing the experimental spectra with *simulations* based on the dual-input ANN output. Remarkably, an excellent agreement was achieved without requiring post-ANN *fitting*. Additionally, a comprehensive comparison was made between single-input and dual-input ANN analysis algorithms concerning accuracy and precision. This evaluation demonstrated that allowing a free spectrum shift in the dual-input ANN training set offers a systematic and more robust analysis approach by reducing susceptibility to inaccurately known setup parameters, therefore providing more reliable results. This marks a major step towards precise analysis methodologies in the study of complex 3D micro- and nanostructures by simultaneously evaluating measurements taken under multiple experimental conditions using a machine learning-based approach^46,47^. Moreover, the multi-input ANN algorithm not only tackles challenges in simultaneous RBS spectrum analysis, as illustrated in this example, but also exhibits great potential for advancing the study of materials across a wide variety of high-throughput experimental techniques probing depth, composition, or chemical properties, whereby the combined analysis of measurements performed under different experimental conditions enhances the accuracy of the results.

## Methods

**Figure 7:**
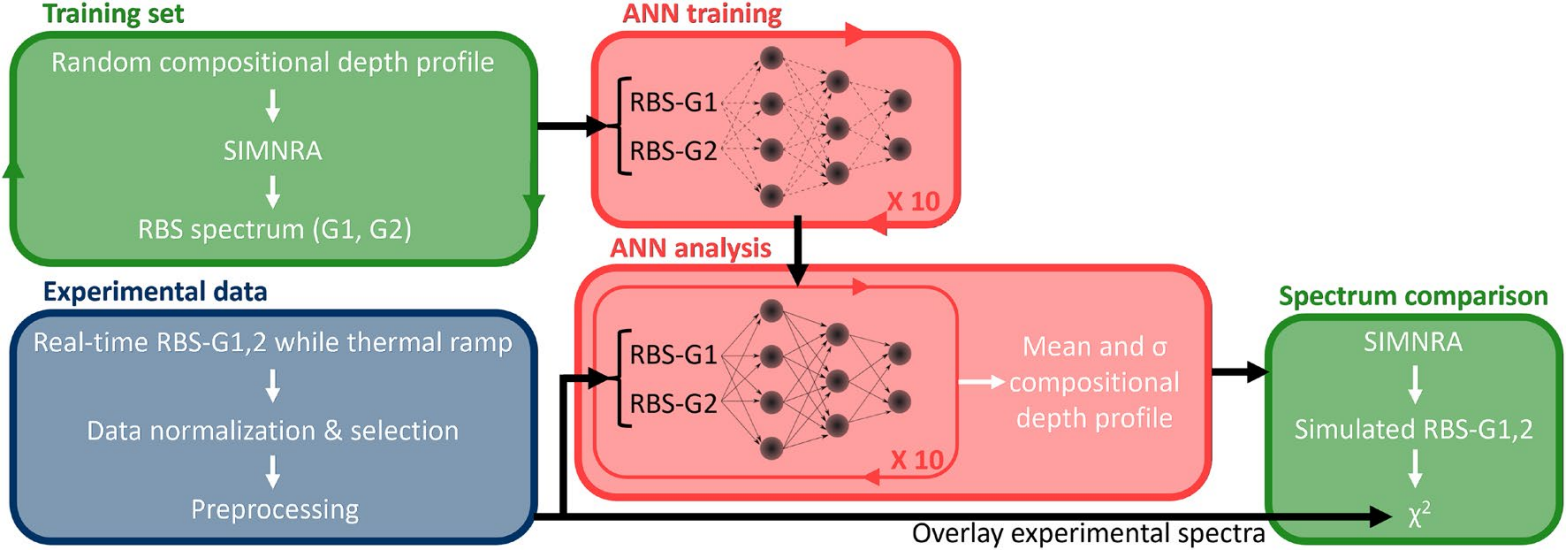
Schematic overview illustrating the DI ANN analysis applied to the real-time RBS data set. The blue box encompasses the experimental data acquisition and preprocessing. The green boxes represent the utilization of SIMNRA including the forward simulation of RBS spectra. The red boxes cover the DI ANN analysis approach.

An overview of the machine learning analysis workflow is shown in Fig. 7.

### Input preprocessing

Initially, a normalization region was defined, spanning channels 250 to 300 (Ge substrate) for both G1 and G2 geometries. Subsequently, the counts in the channels of interest (channels 300 to 500 for both G1 and G2) were normalized to the mean number of counts per channel in the normalization region of the respective geometries, which corresponds to the normalization of the number of incident ions. Subsequently, input counts with values below 0.015 were regarded as background noise and adjusted to zero. Finally, the spectrum was rebinned from 200 channels to 100 channels. This resulted in a feature vector size of 100 for the SI-G1 and SI-G2 ANN analysis, and a feature vector size of 200 for the DI ANN analysis. This data preprocessing did not result in a decreased accuracy of the analysis.

### ANN architecture

The hidden layers in the architecture of the ANNs (low- and high-temperature domain DI, SI-G_1_, SI-G_2_) consist of 200 and 50 nodes which are fully interconnected by a rectified linear unit (ReLU) activation function^48^.

The low-temperature domain ANN (DI, SI-G_1_, SI-G_2_) produces 11 outputs: the areal densities of each of the elements present in the unreacted Ni (1 output), Ni_5_(Ge_1-x_Sn_x_)_3_ (3 outputs), NiGe_1-y_Sn_y_ (3 outputs), and unreacted Ge_1-z_Sn_z_ (2 outputs) layers, and the areal density of the Sn enrichment at the surface (1 output) and at the interface between the NiGe_1-y_Sn_y_ and the unreacted Ge_1-z_Sn_z_ layer (1 output). The high-temperature domain ANN (DI) produces a total of 8 outputs: the areal densities of the elements in the NiGe_1-y_Sn_y_ (3 outputs) and unreacted Ge_1-z_Sn_z_ layers (2 outputs), the areal density of the Sn interface enrichment (1 output), and the areal density and roughness of the Sn surface layer (2 outputs). The latter allows the modeling of Sn surface precipitation, which was observed after thermal annealing at 550 °C using scanning electron microscopy^32^. A thickness distribution following a Gamma function with a small mean layer thickness $\bar{d}$ and large standard deviation $\sigma$ ($\sigma ~\ge ~\bar{d}$) enables to address the precipitation-induced changes in the RBS spectrum^49^. In this approximation, correlation effects between film roughness and interface crossings of the incident and scattered ions are neglected. This is valid for non-grazing incidence and large scattering angles, hence applicable in this particular case.

To all outputs $Y_i$ variance scaling $Y_i/\sigma$ was applied, with $\sigma$ being the standard deviation of the output feature in the normally distributed training set.

### Training set and training process

The training process sets the weights and biases of all interconnections, aiming to minimize the prediction error on the training set. This training set consisted of patterns of randomly selected compositional depth profiles and the corresponding RBS spectra in scattering geometries G_1_ and G_2_. The distribution boundaries of the sample structure and setup parameters in the training set define the parameter space of the ANN, which should cover the entire experimental parameter space. The training set of the low-temperature domain DI ANN consisted of 150,000 patterns within the defined parameter space including a variable total Ni areal density, a roughness of the Ni surface layer, a fixed stoichiometry of the Ni_5_(Ge_1-x_Sn_x_)_3_, NiGe_1-y_Sn_y_, and Ge_1-z_Sn_z_ layers, and free Sn fractions *x*, *y*, *z*. All free parameters were randomly selected from a normal distribution. The training set of the high-temperature domain DI ANN consisted of 50,000 patterns within the defined parameter space, including a variable Ni areal density, a fixed stoichiometry of the NiGe_1-y_Sn_y_ and Ge_1-z_Sn_z_ layers, a random Sn fraction *y*, *z*, a Sn surface layer with extreme roughness to resemble the surface precipitation, and a random areal density of the Sn interface layer. In both training sets, the energy calibration offset of each scattering geometry spectrum was a free parameter, aiming to cover spectrum shifts occurring during the real-time run.

The supervised learning of the ANN requires the forward simulation of RBS spectra. Multiple software implementations enable the calculation of the spectra based on the physics of the interaction of an ion beam with matter. A comparative study assessing the quantitative and qualitative aspects of these simulation codes, along with a quantitative comparison of the analysis of experimental spectra was conducted by the International Atomic Energy Agency^25^. From this, it was concluded that the analysis of experimental spectra using the new generation codes SIMNRA^39^ and NDF^50^ demonstrates excellent agreement amongst the codes. The consistent performance, encompassing spectrum generation time and precision, together with the ability to generate a large number of spectra has led to the frequent use of these software implementations in single-input ANN analysis applications for RBS data^12,30,38^. It is essential to note that, regardless of the forward simulation software employed, the overall uncertainty of the analysis is influenced by both the code uncertainty and the uncertainties associated with the parameters utilized in the simulation. These parameters include the electronic stopping power (used from the SRIM 2003 stopping power database^51^) and the scattering cross sections. Given the usability and good documentation of SIMNRA, the decision was made to employ this forward simulation software for the generation of the training spectra, with the subsequent addition of Poisson statistics to mimic experimental spectra.

The training process was executed in Matlab using the Adam optimizer with 1000 epochs. For the adaptive moment estimation, a gradient decay factor of 0.900 and a squared gradient decay factor of 0.999 were used. The initial learning rate was 0.001 followed by a learn rate drop factor of 0.1 for a drop period of 10 epochs. L2 regularization was included, through the addition of a penalty term with a regularization hyperparameter ($\lambda$) of $10^{-4}$ to the least-squares loss function to avoid overfitting. Parity plots were generated using a designated test set of 15,000 patterns, selected and excluded from the training set, to compare the actual areal density to the areal density predicted by ANN analysis. The linear correlation between the actual and predicted values, and the comparable root-mean-square error of the training and test set confirm the successful training and predictive capability.

## Data Availability

The data that support the findings of this study are available from the corresponding author upon reasonable request.
